# A multiple distributed representation method based on neural network for biomedical event extraction

**DOI:** 10.1186/s12911-017-0563-9

**Published:** 2017-12-20

**Authors:** Anran Wang, Jian Wang, Hongfei Lin, Jianhai Zhang, Zhihao Yang, Kan Xu

**Affiliations:** School of Computer Science and Technology, Dalian University of Technology, Dalian, China

**Keywords:** Biomedical event extraction, Distributed representation, Deep learning, Convolutional neural network

## Abstract

**Background:**

Biomedical event extraction is one of the most frontier domains in biomedical research. The two main subtasks of biomedical event extraction are trigger identification and arguments detection which can both be considered as classification problems. However, traditional state-of-the-art methods are based on support vector machine (SVM) with massive manually designed one-hot represented features, which require enormous work but lack semantic relation among words.

**Methods:**

In this paper, we propose a multiple distributed representation method for biomedical event extraction. The method combines context consisting of dependency-based word embedding, and task-based features represented in a distributed way as the input of deep learning models to train deep learning models. Finally, we used softmax classifier to label the example candidates.

**Results:**

The experimental results on Multi-Level Event Extraction (MLEE) corpus show higher F-scores of 77.97% in trigger identification and 58.31% in overall compared to the state-of-the-art SVM method.

**Conclusions:**

Our distributed representation method for biomedical event extraction avoids the problems of semantic gap and dimension disaster from traditional one-hot representation methods. The promising results demonstrate that our proposed method is effective for biomedical event extraction.

## Background

With the exponentially increasing use of biomedical data, manually extracting useful information from massive biomedical text and associating them becomes imperative. As one of the information extraction areas, biomedical event extraction aims to extract more fine-grained and complex biomedical relations between entities such as biological molecules, cells, and tissues from texts and plays an important role in biomedical research [1].

A biomedical event is normally described by the type of event, a trigger and arguments. The goal of extraction is to detect the type of trigger and furthermore to detect the relation type between triggers with arguments. There are two common approaches on biomedical event extraction: rule-based and machines learning (ML)-based approaches. The rule-based approach builds up the model based on a dictionary and patterns generated from annotated events. Therefore, the model has a high precision on prediction but a low recall because of the poor generalization ability from stable rules. While ML-based approaches show a nice generalization ability on the recent Biomedical tasks [2]. The common pipeline for event extraction includes two subtasks: event trigger identification and event argument detection from documents that already contain Named Entity Recognition (NER). Each task can be regarded as a classification problem. The ML approaches, especially using SVM, have reached the best performance in recent BioNLP-ST tasks of the Genia Event Extraction (GE) [2–7].

The main advantage of the ML-based approach is that rich features can be extracted from text. However, the wide array of defined features may cause difficulties. Additional complex features engineering are needed when applying this model to different biomedical event extraction tasks. Moreover, the hand designed features are mostly one-hot features which are restricted by the problems of semantic gap and dimension disaster. On the other hand, the deep learning methods have achieved considerable progress after word2Vec [8], an effective tool of word embedding representation proposed on some natural language processing tasks such as named entity recognition [9] and sentence classification [10].

To decrease the complexity of feature engineering and detect underlying semantic relations among features automatically, we proposed a distributed representation method which contains not only word embedding information but also task-based distributed features like trigger type on deep learning models to realize event trigger identification and event argument detection to complete biomedical event extraction. Our method was applied on MLEE corpus [1]. This corpus was manually annotated by the guideline formalized in the BioNLP Shared Tasks on event extraction. Our method achieved a higher F-score on both trigger identification and event extraction compared to the baseline [3]. The results demonstrate that the proposed method is effective for biomedical event extraction.

## Methods

**Fig. 1:**
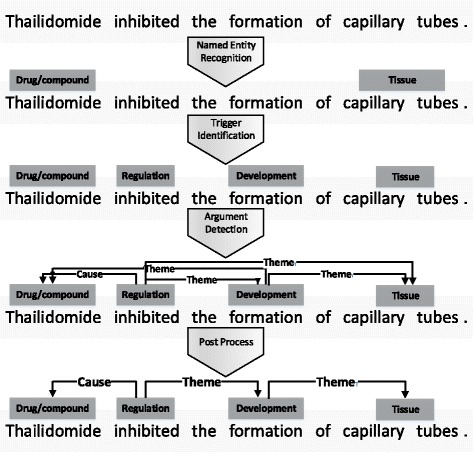
The procedure of biomedical event extraction

As mentioned before, biomedical event extraction is to extract complex biomedical relations between biomedical entities. To simplify the task, the common work flow divides the task into some subtasks shown in Fig. 1. As NER is a mature field in NLP study, the corpus, which followed the guidelines of BioNLP Shared Tasks for biomedical event extraction, are already annotated with biomedical entities to decrease the complexity of this task. With the given entities and context information, triggers and arguments need to be detected to form biomedical events. After some post process with rules designed to filter out incorrect relations, biomedical event extraction is completed.

Our main work focuses on using the deep learning model with the distributed representation method on trigger and arguments detection to improve the accuracy of biomedical event extraction.

To let the model fully learn from training data, we describe each sample in two aspects. First, we utilize word embedding to form the basic representation of context. We parsed all available PubMed abstracts with Gdep [11] to get the contexts of dependency and fed the context from the dependency tree to word2vecf [12] to acquire word embedding with stronger semantic relation. Second, we extended the representation with other task-based features’ distributed representation like Part-of-speech (POS), distance which achieved positive results in previous works [3] to supplement sentence structure information or information that needs to be reinforced.

We discussed the distributed representation of two subtask problems and deep learning models in the follow sections in detail.

### Event trigger identification

The main problem of trigger identification is to predict the trigger type of every word in a sentence. This can be represented as a classification problem shown as follow:

1 $$ \mathcal{F}\left(\uppsi \left({\mathrm{w}}_{\mathrm{i}}\right)\right)=\Big\{\kern0.2em {\displaystyle \begin{array}{c}\mathcal{C}\kern1em \mathrm{Positive},\mathcal{C}\kern0.2em \mathrm{is}\  \mathrm{the}\  \mathrm{type}\ \mathrm{id}\ \mathrm{of}\  \mathrm{trigger}\\ {}-1\kern1em \mathrm{Negative}\kern11.9em \end{array}}\operatorname{} $$

**Fig. 2:**
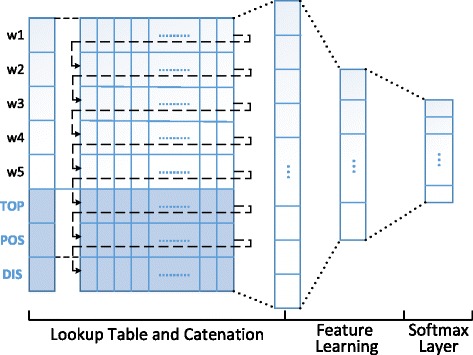
The model of trigger identification

Where ψ(w_i_) denotes distributed representation of each word, and $\mathcal{F}$ is a classification function. The model we have chosen is the deep neural network, the framework is shown as Fig. 2.

#### Multiple distributed representation construction

A sentence might contain more than one event, but most events will be contained in a single sentence. To decrease the complexity of the model, we divided corpus into sentences and built feature representations from sentence information. Inspired by the language model, we drew the basic picture of trigger candidates by their context and expanded details by their POS. Due to the deep relevance between triggers and entities (participate candidates), we utilized the distance between them to describe the possibility of the candidate to be a trigger. Moreover, we added sentence topic probability to discriminate sentences.

- Context representation

As mentioned before, we adopted the context words between candidate triggers to predict trigger type. Given a sentence S = …w_i − 2_w_i − 1_T_i_w_i + 1_w_i + 2_…, where T_i_denotes trigger to be predicted, and assuming the window size (d_win_) is 2, therefore words w_i − 2_,  w_i − 1_,  w_i + 1_,  w_i + 2_ are what we will adopt.

First, we found words around the trigger to be predicted from a dictionary of training set according to d_win_, then we initialized these words’ vectors according to the dependency-based word embedding table. If the window is out of the edge of the sentence or can’t be found in the word embedding table, then we initialize the embedding in a random way. Finally, we concatenated them as follows:

2 $$ {\uppsi}_{\mathrm{con}}\left({\mathrm{T}}_{\mathrm{i}}\right)=\left[\right\langle \mathrm{W}\left\langle {}_{\left[{\mathrm{w}}_{\mathrm{i}-{\mathrm{d}}_{\mathrm{w}\mathrm{in}}}\right]}\cdot \cdots \cdot \right\langle \mathrm{W}\left\langle {}_{\left[{\mathrm{w}}_{\mathrm{i}-1}\right]}\cdot \right\langle \mathrm{W}\left\langle {}_{\left[{\mathrm{T}}_{\mathrm{i}}\right]}\cdot \right\langle \mathrm{W}\left\langle {}_{\left[{\mathrm{w}}_{\mathrm{i}+1}\right]}\cdots \cdot \right\langle \mathrm{W}\left\langle {}_{\left[{\mathrm{w}}_{\mathrm{i}+{\mathrm{d}}_{\mathrm{w}\mathrm{in}}}\right]}\right] $$

Where $\mathrm{W}\in {\mathcal{R}}^{\dim \times \left|\mathcal{D}\right|}$ represents the dependency-based word embedding table, dim is the dimensionality of word embedding we trained in the previous work, and $\left|\mathcal{D}\right|$ is the size of the dictionary.

- Topic representation

Ambiguity is a problem that can’t be neglected in word classification tasks. A word can express various meanings according to the specific context. Therefore, disambiguation plays an important role in word classification tasks including trigger identification.

Here we added the topic feature to represent sentence topic information of each trigger in the sentence. We used a Latent Dirichlet Allocation (LDA) [13] tool provided by Gensim, a python library, to acquire the topic distribution of every word in a sentence (the probability of a word belonging to one topic), and then multiply the topic probability of every word in one sentence to get the approximate sentence topic distribution [14]. The sentence topic feature is computed by the following equation:

3 $$ {\uppsi}_{\mathrm{top}}\left(\mathrm{S}\right)={\prod}_{\mathrm{j}=\mathrm{i}-{\mathrm{d}}_{\mathrm{w}\mathrm{in}}}^{\mathrm{i}+{\mathrm{d}}_{\mathrm{w}\mathrm{in}}}{\left\langle {\mathrm{W}}_{\mathrm{top}}\right\rangle}_{\left[{\mathrm{w}}_{\mathrm{j}}\right]} $$

Where $\left\langle {\mathrm{W}}_{\mathrm{top}}\right\rangle \in {\mathcal{R}}^{{\mathrm{d}}_{\mathrm{top}}\times \mid \mathrm{D}\mid }$ is words topic distribution, d_top_ represents the total number of topics, and ∣ D∣ denotes the size of the dictionary.

- POS representation

The POS of triggers are mostly related to verbs which makes them a considerable role in trigger identification. The distributed representation of POS is shown as follow:

4 $$ {\uppsi}_{\mathrm{pos}}\left({\mathrm{T}}_{\mathrm{i}}\right)={\left\langle {\mathrm{W}}_{\mathrm{pos}}\right\rangle}_{\left[{\mathrm{pos}}_{{\mathrm{T}}_{\mathrm{i}}}\right]} $$

Where $\left\langle {\mathrm{W}}_{\mathrm{pos}}\right\rangle \in {\mathcal{R}}^{{\mathrm{d}}_{\mathrm{pos}}\times \left|{\mathcal{D}}_{\mathrm{pos}}\right|}$ represent POS vector table, $\left|{\mathcal{D}}_{\mathrm{pos}}\right|$ is the number of different POS types in the training set, and d_pos_ is the dimensionality of the POS vector. W_pos_ is initialized at random and updated while training.

- Distance representation

Triggers have a deep relevance with the entities. The longer the distance between the word and the entities, the lower the possibility it can be a trigger. Based on our previous work [15], we adopt the distance between the trigger and entities in the dependency tree, and the distance is shown as follows:

5 $$ {\uppsi}_{\mathrm{dis}}\left({\mathrm{T}}_{\mathrm{i}}{\mathrm{E}}_{\mathrm{j}}\right)={\left\langle {\mathrm{W}}_{\mathrm{dis}}\right\rangle}_{\left[\mathrm{dis}\left({\mathrm{T}}_{\mathrm{i}},{\mathrm{E}}_{\mathrm{j}}\right)\right]} $$

Where dis(T_i_, E_j_) represents the distance of the candidate trigger to the closest entities, and $\left\langle {\mathrm{W}}_{\mathrm{d}\mathrm{is}}\right\rangle \in {\mathcal{R}}^{{\mathrm{d}}_{\mathrm{d}\mathrm{is}}\times \left|{\mathcal{D}}_{\mathrm{d}\mathrm{is}}\right|}$ is the distance table. $\left|{\mathcal{D}}_{\mathrm{dis}}\right|$ is the number of distance possibilities and d_dis_ is the dimensionality of the distance vector. W_dis_ is initialized at random and updated while training.

Finally, the concatenation of these four feature representations formed the distributed representation of candidate triggers to be the input layer fed to the deep learning model.

6 $$ \uppsi \left({\mathrm{T}}_{\mathrm{i}}\right)=\kern0.5em {\uppsi}_{\mathrm{con}}\left({\mathrm{T}}_{\mathrm{i}}\right)\bullet {\uppsi}_{\mathrm{top}}\left(\mathrm{S}\right)\bullet {\uppsi}_{\mathrm{pos}}\left({\mathrm{T}}_{\mathrm{i}}\right)\bullet {\uppsi}_{\mathrm{dis}}\left({\mathrm{T}}_{\mathrm{i}}{\mathrm{E}}_{\mathrm{j}}\right) $$

Where ∙ denotes concatenation.

#### Deep learning model

We fed ψ(*T*_*i*_) to the deep learning model provided by Theano [16] and sped up computation by running on the GPU. To optimize the learning model, we utilized Adadelta [17] to adjust the learning rate automatically and used Dropout [18] to prevent the model from over fitting. The parameters were set as in Table 1.

**Table 1:** The setting of parameters in neural network for event trigger identification

	Word embedding dimension	Hidden layers	Hidden-layer notes	Batch	Dropout Rate
Value	100	3	1000	512	0.2

### Event argument detection

To extract the event, we need to detect event arguments after trigger identification. This step is for examining whether entities have any relation with the triggers and detecting the type of relation. In the end, an event can be generated from the composition of a trigger and its event arguments. The process can be represented as a classification problem and modeled as follows:

7 $$ \mathcal{F}\left(\uppsi \right){T}_i\left({E}_j|{T}_k\right)\left)\right)=\Big\{\kern0.2em {\displaystyle \begin{array}{c}\mathcal{C}\kern1.5em \mathrm{Positive},\mathcal{C}\kern0.2em \mathrm{is}\  \mathrm{the}\  \mathrm{type}\ \mathrm{id}\ \mathrm{of}\  \mathrm{event}\  \mathrm{argument}\\ {}-1\kern1.5em \mathrm{Negative}\kern11.9em \end{array}}\operatorname{} $$

Where ψ denotes the feature extraction function, T_i_(E_j_| T_k_) denotes the relation of entity E_j_ with trigger T_k_ (E_j_ can be an argument or a trigger).

**Fig. 3:**
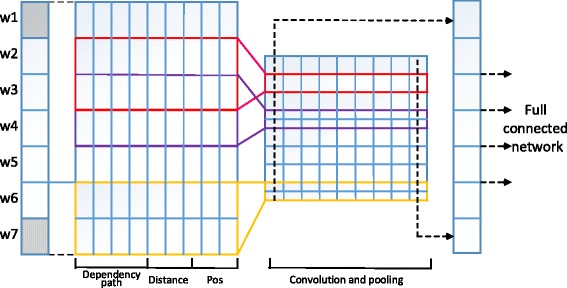
The model of argument detection based on convolutional neural network

Some previous works showed that convolutional neural network (CNN) has significant effectiveness in the modeling of sentences [10]. The ability of gaining feature maps by different filters helps to exploit rich and deep features. Also CNN can unify the length from various distances between triggers and participate candidates. Therefore, we employed CNN to model the sentence based on the dependency path between the trigger and candidates to detect trigger-argument or trigger-trigger relation. The framework is shown as Fig. 3.

#### Multiple distributed representation construction

To detect the relation of triggers and arguments, we utilized the words between them as the fundamental representation of their relations. We also added POS and distance to describe words on the dependency path more sufficiently. In addition, we used the type of trigger and argument to express their type of relation.

- Dependency-path representation

Traditional methods in relation classification tasks use words before two entities to represent their relation. However due to the high frequency of long and complex sentences in biomedical corpus, it is hard to detect semantic relation between trigger and arguments. To solve that, we adopted the word on the dependency-path between the trigger and candidates to represent the logical relation of them.

After parsing a sentence, we get the dependency path S = w_1_w_2_w_3_w_4_w_5_…w_n_ between trigger and entities. We then convert the sequence to a matrix according to the word embedding:

8 $$ {\uppsi}_{\mathrm{con}}\left(\mathrm{S}\right)=\left[\begin{array}{ccc}{{\left\langle \mathrm{W}\right\rangle}_{\left[{\mathrm{w}}_1\right]}}^1& \cdots & {{\left\langle \mathrm{W}\right\rangle}_{\left[{\mathrm{w}}_{\mathrm{n}}\right]}}^1\\ {}\vdots & \ddots & \vdots \\ {}{{\left\langle \mathrm{W}\right\rangle}_{\left[{\mathrm{w}}_1\right]}}^{\mathrm{d}}& \cdots & {{\left\langle \mathrm{W}\right\rangle}_{\left[{\mathrm{w}}_{\mathrm{n}}\right]}}^{\mathrm{d}}\end{array}\right] $$

Where $\left\langle \mathrm{W}\right\rangle \in {\mathcal{R}}^{\mathrm{d}\times \left|\mathcal{D}\right|}$ represents word embedding table as mentioned before.

- POS representation

Different from POS representation in trigger identification, we utilized POS from words on the dependency path:

9 $$ {\psi}_{pos}(S)=\left[\begin{array}{ccc}{{\left\langle {W}_{pos}\right\rangle}_{\left[ pos\Big({w}_{1\Big)}\right]}}^1& \cdots & {{\left\langle {W}_{pos}\right\rangle}_{\left[ pos\Big({w}_{\mathrm{n}\Big)}\right]}}^1\\ {}\vdots & \ddots & \vdots \\ {}{{\left\langle {W}_{pos}\right\rangle}_{\left[ pos\Big({w}_{1\Big)}\right]}}^d& \cdots & {{\left\langle {W}_{pos}\right\rangle}_{\left[ pos\Big({w}_{\mathrm{n}\Big)}\right]}}^d\end{array}\right] $$

Where $\left\langle {\mathrm{W}}_{\mathrm{pos}}\right\rangle \in {\mathcal{R}}^{{\mathrm{d}}_{\mathrm{pos}}\times \left|{\mathcal{D}}_{\mathrm{pos}}\right|}$ represents POS embedding table, which is initialized at random and updated while training.

- Distance representation

There are two distances we measured to capture the relative distance. One is the distance from word *w*_i_ in *S* to the first entity (or trigger), the other one is from *w*_i_ to the second entity (or trigger) shown as follows respectively:

10 $$ \kern9em {\uppsi}_{\mathrm{d}\mathrm{is}\_\mathrm{e}1}\left(\mathrm{S}\right)=\kern2.5em \left[\begin{array}{ccc}{{\left\langle {\mathrm{W}}_{\mathrm{d}\mathrm{is}}\right\rangle}_{\left[\mathrm{dis}\left({\mathrm{w}}_1,{\mathrm{e}}_1\right)\right]}}^1& \cdots & {{\left\langle {\mathrm{W}}_{\mathrm{d}\mathrm{is}}\right\rangle}_{\left[\mathrm{dis}\left({\mathrm{w}}_{\mathrm{n}},{\mathrm{e}}_1\right)\right]}}^1\\ {}\vdots & \ddots & \vdots \\ {}{{\left\langle {\mathrm{W}}_{\mathrm{d}\mathrm{is}}\right\rangle}_{\left[\mathrm{dis}\left({\mathrm{w}}_1,{\mathrm{e}}_1\right)\right]}}^{{\mathrm{d}}_{\mathrm{d}\mathrm{is}}}& \cdots & {{\left\langle {\mathrm{W}}_{\mathrm{d}\mathrm{is}}\right\rangle}_{\left[\mathrm{dis}\left({\mathrm{w}}_{\mathrm{n}},{\mathrm{e}}_1\right)\right]}}^{{\mathrm{d}}_{\mathrm{d}\mathrm{is}}}\end{array}\right] $$

11 $$ \kern4.75em {\psi}_{dis\_e2}(S)=\kern2.5em \left[\begin{array}{ccc}{{\left\langle {W}_{dis}\right\rangle}_{\left[ dis\left({w}_1,{e}_2\right)\right]}}^1& \cdots & {{\left\langle {W}_{dis}\right\rangle}_{\left[ dis\left({w}_n,{e}_2\right)\right]}}^1\\ {}\vdots & \ddots & \vdots \\ {}{{\left\langle {W}_{dis}\right\rangle}_{\left[ dis\left({w}_1,{e}_2\right)\right]}}^{d_{dis}}& \cdots & \kern0.2em {{\left\langle {W}_{dis}\right\rangle}_{\left[ dis\left({w}_n,{e}_2\right)\right]}}^{d_{dis}}\end{array}\right] $$

Where, $\left\langle {\mathrm{W}}_{\mathrm{d}\mathrm{is}}\right\rangle \in {\mathcal{R}}^{{\mathrm{d}}_{\mathrm{d}\mathrm{is}}\times \left|{\mathcal{D}}_{\mathrm{d}\mathrm{is}}\right|}$ represents the distance embedding table, which is initialized at random and updated while training.

- Type representation

According to the domain knowledge of the corpus, we know that there are many constant type combinations such as entity “Cell” that are more likely to have a relation to the event type “Cell proliferation”. To describe the probability of the relation two entities have, we combine the type of triggers or entities with dependency-path word representation to describe the sentence feature.

12 $$ {\psi}_{type}(S)=\left[\begin{array}{cc}{{\left\langle {W}_{type}\right\rangle}_{\left[{e}_1\right]}}^1& {{\left\langle {W}_{type}\right\rangle}_{\left[{e}_2\right]}}^1\\ {}\vdots & \vdots \\ {}{{\left\langle {W}_{type}\right\rangle}_{\left[{e}_1\right]}}^{d_{type}}& {{\left\langle {W}_{type}\right\rangle}_{\left[{e}_2\right]}}^{d_{type}}\end{array}\right] $$

Where $\left\langle {\mathrm{W}}_{\mathrm{type}}\right\rangle \in {\mathcal{R}}^{{\mathrm{d}}_{\mathrm{type}}\times \left|{\mathcal{D}}_{\mathrm{type}}\right|}$ represents the entities type table, which is initialized at random and updated while training as well. $\left|{\mathcal{D}}_{\mathrm{type}}\right|$ is the total type number, and d_type_ is the dimension of each type which should be the same as word embedding. For the type and dependency words all represent sentence feature and we concatenate them as one feature.

Finally, we map these four distributed representations as the description of sentences and employ CNN to build the classification model.

13 $$ \psi (S)=\left[\begin{array}{c}{\psi}_{con}(S)\bullet {\psi}_{type}(S)\\ {}{\psi}_{pos}(S)\\ {}{\psi}_{dis\_e1}(S)\\ {}{\psi}_{dis\_e2}(S)\end{array}\right] $$

#### Convolutional neural network

The main distinguished layers of CNN from other neural network are convolutional layer and pooling layer. The filters, provided in the convolutional layer, have a small receptive field and extend through all input volume with shared parameters to learn the various levels of features from the input layer. We set three filters based on our sentence length of corpus to extract rich features. The pooling layer helps to progressively reduce the size of the representation to reduce the amount of parameters and computation in the network. We adopted max-pooling to extract the most valuable feature representation. The full connected network is similar to the model mentioned above. The parameters were set as in Table 2.

**Table 2:** The parameters of convolutional neural network for event argument detection

	Word embedding dimension	Filters	Hidden-layer notes	Batch	Dropout
Value	50	[3, 5, 7]	1000	128	0.2

## Results and discussion

### Datasets and evaluation metrics

We evaluated our proposed method on MLEE corpus, which spanned all levels of biomedical organization and covered 19 types of triggers. The details of the dataset is shown in Table 3.

**Table 3:** The statistics of MLEE dataset

Data	Train	Validation	Test	All
Document	131	44	87	262
Sentence	1271	457	880	2608
Word	27,875	9610	19,103	56,588
Entity	4147	1431	2713	8291
Event	3296	1175	2206	6677

In an event extraction task, there are three parts that need to be equal when identifying if two events are equal: event type, event trigger, and argument. In this paper we adopted two methods from BioNLP-ST task to evaluate identity, the approximate span matching method to evaluate if the predicted trigger is equal to the annotated trigger and the approximate recursive matching method. This was to judge if two events are equal. Further, we measured the performance of our proposed method by F-score, precision and recall.

### Experimental results

To evaluate our proposed method in this paper, we compare the performance of the two baselines constructed by Zhou [3], which are state-of-the-art methods based on the MLEE corpus as well as using SVM. Then we tested different feature combinations to detect the contribution from different features. Furthermore, we applied our method on datasets of GE task in BioNLP-ST2009, 2011, 2013 as well to inspect the generalization ability of the method.

- Performance comparison with other methods

We compared our method with the two state-of-the-art methods on trigger identification and event extraction in Tables 4 and 5. Based on the results, we achieved a better F-score performance on both trigger identification and event extraction. The result indicates that the proposed distributed representation, including dependent context which was formed by word embedding and task-based features on the deep learning methods, works well on biomedical event extraction tasks compared to the baseline approach using SVM with manually complex features. The lower recall might be caused by, insufficiency of the training data from event types appearing too seldom to be learned, or different post process rules.

- Feature analysis

**Table 4:** The comparison of results on trigger identification in MLEE

Method	F-score (%)	Precision (%)	Recall (%)
Proposed method	77.97	80.92	75.23
Zhou [3]	76.89	72.17	82.26
Pyysalo [1]	75.84	70.79	81.69

**Table 5:** The comparison of results on events extraction in MLEE

Method	F-score (%)	Precision (%)	Recall (%)
Proposed method	58.31	60.56	56.23
Zhou [3]	57.41	55.76	59.16
Pyysalo [1]	55.20	62.28	49.56

The distributed representation of NLP tasks are mostly based on context information. In Tables 6 and 7, we listed the feature combination performance on F-score, precision, and recall to analyze each feature’s contribution.

**Table 6:** The comparison of results with different features on trigger identification

Method	F-score (%)	Precision (%)	Recall (%)
context	75.51	78.83	72.47
context +topic	75.96	79.88	72.42
context +POS	75.98	79.84	72.47
context +distance	77.27	80.60	74.18
context + topic+ POS + distance	77.97	80.92	75.23

**Table 7:** The comparison of results with different features on events extraction

Method	F-score (%)	Precision (%)	Recall (%)
context	55.60	58.34	53.10
context +POS	56.24	59.28	53.49
context +distance	57.68	60.04	55.49
context +type	57.40	59.21	55.69
context +POS + distance + type	58.31	60.56	56.23

Based on the results from Tables 6 and 7, we can find that distance is a significant feature for trigger identification, beside context, which verifies the deep relevance between entities (arguments) and triggers. Also domain knowledge “type” in Table 7 is shown to have certain contributions to the performance.

All the extra features we added on context are the main features in SVM, which indicates that manually extracted features still contribute to deep learning models either by extending features not learned by the model or by reinforcing the features already learned by model.

- Generalization assessment

To examine our proposed method’s generalization ability on other biomedical corpus, we applied our method on the latest BioNLP event extraction tasks as well. The baselines we chose are distinguished event extraction tools that emerged in recent years and achieved outstanding performance in the tasks respectively. EvenMine [4] and UMASS [5] yield better results than the methods in the evaluation of BioNLP-ST’09. FAUST [2] is a model based on UMASS and achieved the best performance in BioNLP-ST’11. EVEX [6]. This method is ranked first in BioNLP-ST’13 and uses the output of the TEES system as features of confidence scores from other models and added extra sentence structure information features for training on SVM.

Tables 8, 9, 10 demonstrate that our proposed deep learning model based on basic pipeline of biomedical event extraction already achieved comparative results to all the SVM methods on all three datasets. From previous experience, the more complex a SVM model becomes the higher its performance. The latest SVM methods focus more on model combination and added task-based rules to improve the performance on specific evaluation task dataset. On the other hand, the amount of training data decide if a deep learning model can learn fully. However, our proposed method reached the same level of the best performance of evaluation tasks only with basic work flow and few task-based features which indicates the operation simplicity and generalization ability of our method.

**Table 8:** The comparison with the best performance of event extraction results in BioNLP-ST2009 dataset

Method	F-score (%)	Precision (%)	Recall (%)
Proposed method	59.94	64.34	56.10
EventMine [4]	58.81	63.17	55.00
UMASS [5]	58.70	–	–

**Table 9:** The comparison with the best performance of event extraction results inBioNLP-ST2011 dataset

Method	F-score (%)	Precision (%)	Recall (%)
FAUST [2]	55.90	–	–
Proposed method	55.20	58.33	52.38
UMASS [2]	54.80	–	–

**Table 10:** The comparison with the best performance of event extraction results in BioNLP-ST2013 dataset

Method	F-score (%)	Precision (%)	Recall (%)
EVEX [6]	50.97	58.03	45.44
TEES-2.1 [7]	50.74	56.32	46.17
Proposed method	50.12	55.67	45.58

## Conclusion and future work

In this paper, we have presented a multiple distributed representation method which combines dependent context formed by word embedding with task-based features from biomedical text and fed it to deep learning models to achieve biomedical event extraction. This method avoids the problems of semantic gap and dimension disaster from traditional one-hot representation methods and achieved a promising result on several datasets using the basic pipeline of event extraction with few extra manually extracted features instead of complex feature engineering. In particular, the distributed representation can be extended as the supplement of word embedding depending on the needs of specific tasks. Besides the semantic information from word embedding, the model can extend task-based representation according to the domain knowledge, which makes the model more flexible for different topic based biomedical event extraction tasks.

In the future we plan to modify our deep learning models with attention method. Also we can apply our work on a Recurrent Neural network to learn sequence features and then combine them with the CNN model to enrich the features of the learning model.
